# Electronic transmittance phase extracted from mesoscopic interferometers

**DOI:** 10.1186/1556-276X-7-568

**Published:** 2012-10-13

**Authors:** M Ţolea, V Moldoveanu, IV Dinu, B Tanatar

**Affiliations:** 1, National Institute of Materials Physics, P. O. Box MG-7, Bucharest-Magurele 77125, Romania; 2 Department of Physics, Bilkent University, Bilkent, Ankara 06800, Turkey

**Keywords:** Phase measurement, Aharonov-Bohm interferometers, Phase lapse problem

## Abstract

The usual experimental set-up for measuring the wave function phase shift of electrons tunneling through a quantum dot (QD) embedded in a ring (i.e., the transmittance phase) is the so-called ‘open’ interferometer as first proposed by Schuster et al. in 1997, in which the electrons back-scattered at source and the drain contacts are absorbed by additional leads in order to exclude multiple interference. While in this case one can conveniently use a simple two-path interference formula to extract the QD transmittance phase, the open interferometer has also a number of draw-backs, such as a reduced signal and some uncertainty regarding the effects of the extra leads. Here we present a meaningful theoretical study of the QD transmittance phase in ‘closed’ interferometers (i.e., connected only to source and drain leads). By putting together data from existing literature and giving some new proofs, we show both analytically and by numerical simulations that the existence of phase lapses between consecutive resonances of the ‘bare’ QD is related to the signs of the corresponding Fano parameters - of the QD + ring system. More precisely, if the Fano parameters have the same sign, the transmittance phase of the QD exhibits a *Π* lapse. Therefore, closed mesoscopic interferometers can be used to address the ‘universal phase lapse’ problem. Moreover, the data from already existing Fano interference experiments from Kobayashi et al. in 2003 can be used to infer the phase lapses.

## Background

The phase of the wave function (of an electron, for instance) is a pure quantum mechanical property, without a direct correspondence in classical physics. The phase coherence actually lies in the very definition of mesoscopic physics and plays a key role in phenomena such as quantum interference or bonding of molecular orbitals. Another reason for the increasing interest in the phase problem is a number of intriguing, and so far unexplained, results of the phase-measuring experiments, such as the phase lapse problem (of the transmittance phase between the resonances of a quantum dot, QD) which was called by some authors ‘the longest standing puzzle in mesoscopic physics’ (see e.g., [1]).

Although they are probably obvious for most readers, we consider it useful (for the sake of completeness) to start by briefly defining some terms commonly used in the mesoscopic phase measurement problem: 

1. Transmittance phase: In the plane wave scattering image, the wave function of an incoming electron can be considered to be *e*^ikr^ and it changes to *te*^ikr^ after tunneling through a QD; then |*t*|^2^ is the transmittance amplitude and Arg[*t*] is the transmittance phase.

2. Closed/open interferometers: A *closed* interferometer is actually a mesoscopic ring connected to two leads, also named two-terminal interferometer. In contrast, an *open* interferometer has supplementary terminals called base zones with the aim to absorb the electrons scattered at contacts and to forbid multiple encirclements of the ring.

3. The phase lapse problem: The transmittance phase was experimentally found to exhibit phase lapses of *Π* between *any pairs* of resonances [2], which is a puzzling aspect that did not receive a satisfactory explanation. The only exception was presented in the experiment of Avinun-Kalish et al. [3], for a few-electron quantum dot.

4. Fano parameter: If a variable gate potential is applied on a QD that is inserted in one arm of an Aharonov-Bohm (AB) interferometer, the result in conductance would be asymmetric Fano resonances, with the general equation $G\approx {\left(\varepsilon +q\right)}^{2}/(\varepsilon^{2}+1)$, *q* being called the Fano parameter (*ε*is proportional to the dot energy levels and can be varied by an applied gate potential - see ‘Results and discussion’ Section).

In a first attempt to extract the transmittance phase, Yacoby et al. [4] performed transport measurements on a closed interferometer with a QD inserted in one arm, the phase being extracted from a simple two-path interference formula. The main experimental problem, however, with the ‘closed’ set-up is that the electrons encircle the ring more than once, blurring the single-tunneling phase information.

The problem was solved by Schuster et al. [2] who ‘opened’ the interferometer by adding supplementary base zones to absorb the deflected electrons and ideally to ensure just a single interference. In this way, one can measure not only the amplitude of the transmission, but also its phase, which is extracted from the shift of the AB conductance oscillations.

The Friedel sum rule [5] (see also [6-8]) and other simple models, like the 1D double barrier (see Figure 14 in [9]), suggest that the measured phase increases with *Π* on each QD resonance and remains at a constant value between resonances. Surprisingly, the experiment [2] found instead that between any two consecutive conductance peaks, the transmission phase displays a jump (phase lapse) of *Π*. The transmittance phase measurements in open interferometers was taken a step further by Avinun-Kalish et al. [3] who controlled the QD occupancy via a nearby quantum point contact. The QD was first depleted of electrons and then it is gradually filled. For the first few electrons added to the dot (*N* < 10), the authors reported a non-universality of the phase behavior, which are varied on some resonances and between them with *Π* or fractions of *Π*. This regime was called ‘mesoscopic’. In the multi-electron regime, the ‘universal’ phase lapse with *Π* emerges, as obtained in all the previous experiments.

Many theoretical papers used discrete models for the analysis of the transmittance phase (see, e.g., [9-13]), especially for the non-interacting case. Among the first papers addressing theoretically the behavior of the phase were in [10,11], where it was shown that phase lapses between two resonances can be associated with a zero value of the conductance at the corresponding energy, and this happens between consecutive resonances with the same parity.

The experiments by Avinun-Kalish et al. [3] generated a new direction of theoretical research employing sophisticated many-body techniques to explain the phase behavior in the few-electron regime [14-16]. Other authors addressed the crossover from mesoscopic to universal regime [17-19]. The emergence of the universal phase lapses regime was explained by some authors as a ‘population switching’ inside the quantum dot [20,21]. In [22] a set-up consisting of a double-dot mesoscopic ring and a nearby charge detector was proposed. It is shown that the ‘bare dot’ phase evolution can be extracted from the second harmonic of the AB oscillations.

At this point, it is important to stress that even if the open interferometer solves the problem of multiple interferences, it also has a number of important drawbacks from experimental point of view, in comparison with the closed interferometer: a substantially reduced signal and some uncertainties regarding the influence of the base zones themselves on the measured phase. From theoretical point of view, the open interferometer is difficult to be described by a simple Hamiltonian, even if some efforts have been made [23].

Bearing in mind the mentioned drawbacks of the open interferometer, our goal in this paper is to show what phase information can be extracted from closed interferometers. We prove that the sign of Fano parameter *q* carries the important information about the existence (or not) of a phase lapse with *Π* of the ‘bare’ dot transmittance.

The correspondence we shall prove can be used to interpret already existing experimental results that used closed interferometers in the context of the Fano effect, but did not discuss phase implications. The experimental data suggest that, even if the phase lapses are indeed present *almost* between any pair of resonances, they are not actually *universal*; as consecutive out-of phase resonances are also present (see [24,25] where a closed interferometer were used) and between those resonances, the phase should remain constant.

The outline of the paper is as follows: The Methods and Results and discussion Sections contain the analytical and numerical results connecting the sign of the Fano parameter to the phase lapses of the ‘bare’ dot transmittance, and the last Section presents the Conclusions.

## Methods

**Figure 1:**
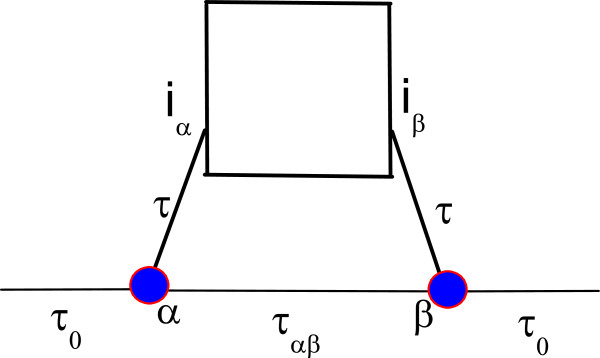
**QD connected to sites*α*** and ***β*and to each other forming a closed interferometer.** Scheme of a QD connected to the sites *α* and *β* connected to each other to form a closed interferometer. The notations are the same as those used in text.

We consider a multi-level quantum dot embedded in an mesoscopic interferometer (see Figure 1). The system will be described by a lattice model, the QD being of arbitrary shape and coupled to the ring in the sites *i*_*α*_ and *i*_*β*_. For simplicity we consider that the ‘ring’ is quite simple and consist of only two sites *α* and *β* connected by the hopping constant *τ*_*αβ*_. Then the Hamiltonian describing the mesoscopic interferometer and the left and right leads coupled to it reads as follows:

(1) $$H=H_{D}+H_{R}+\underset{\gamma =\alpha ,\beta }{\sum }H_{L_{\gamma }}+H_{\mathrm{DR}}+H_{T},$$

where *H*_D_ and *H*_R_ describe the QD (of arbitrary shape, formally a collection of sites with hopping between the nearest neighbors, denoted by the symbol 〈〉) and the ring (consisting just of two sites, interconnected, see Figure 1), while *H*_L__*γ*_, *H*_T_, and *H*_DR_ represent the leads Hamiltonian and the tunneling Hamiltonians, respectively:

(2) $$\begin{array}{lll} \;H_{D} & =\underset{\langle i,j\rangle }{\sum }|i\rangle \langle j|\; & \;\\ \;H_{R} & =\tau_{\text{αβ}}|\alpha \rangle \langle \beta |+\mathrm{h.c.}\; & \;\\ \;H_{L_{\gamma }} & =\underset{k_{L_{\gamma =\alpha ,\beta }}}{\sum }\varepsilon_{k}|k_{L_{\gamma }}\rangle \langle k_{L_{\gamma }}|\; & \;\\ \;H_{T} & =\tau_{0}\underset{k_{L_{\gamma =\alpha ,\beta }}}{\sum }|k_{L_{\gamma }}\rangle \langle \gamma |+\mathrm{h.c.}\; & \;\\ \;H_{\mathrm{DR}} & =\tau (|\alpha \rangle \langle i_{\alpha }|+|\beta \rangle \langle i_{\beta }|)+\mathrm{h.c.}\; \end{array}$$

The diagonal energies for the dot sites and those for the two ring sites have been considered *zero* and have not been written explicitly, for briefness.

## Results and discussion

Let us first briefly review an important result obtained first in [10], for the case of a QD connected directly to leads (and not imbedded in an interferometer). The mentioned result connects the phase evolution between consecutive resonances (more precisely, the existence or not of a phase lapse with *Π*) with the parity of the respective resonances. Then we shall insert the same QD in an interferometer and prove that the information on the resonances parity can be extracted from the sign of the Fano parameter for consecutive Fano lines. We remind here that the parity of a resonance associated to the *n*th state *Ψ*_*n*_ of the dot is defined to be the sign of the product *Ψ*_*n*_(*i*_*α*_)*Ψ*_*n*_(*i*_*β*_). In the absence of Coulomb interaction the conductance is given by the Landauer formula (*E*_F_ is the Fermi level of the leads):

(3) $$G(E_{F})=\frac{2e^{2}}{h}4\Gamma^{2}{\left|G_{i_{\alpha }i_{\beta }}^{D}\right|}^{2},$$

where *G*^D^ is the effective retarded Green function *G*^D^(*E*) = (*E*−*H*^D^ + *Σ*^L^)^−1^, and *Σ*^L^ = *iΓ*(|*i*_*α*_〉〈*i*_*α*_| + |*i*_*β*_〉〈*i*_*β*_|) is the lead’s self-energy with $\Gamma =2\Pi \rho_{0}\tau_{0}^{2}$, *ρ*_0_ being the density of states in the leads. In the above equation, we did not write explicitly the energy dependence of the right hand term and will omit it also in the following sections, whenever the meaning is obvious.

Levy Yeyati and Büttiker [10] approximated the Green function as follows (*E*_*n*_ is the energy of the state *Ψ*_*n*_):

(4) $$G_{i_{\alpha }i_{\beta }}^{D}(E_{F})\approx \underset{n}{\sum }\frac{\Psi_{n}\left(i_{\alpha }\right)\Psi_{n}\left(i_{\beta }\right)}{E_{F}-E_{n}+\mathrm{i\Gamma}[\Psi_{n}^{2}\left(i_{\alpha }\right)+\Psi_{n}^{2}\left(i_{\beta }\right)]},$$

which means that, in the limit of low coupling, the Green function can be written as a sum of resonances, and furthermore, the parameters of interest have simple expressions. Now the question was whether the above formula contains information about the phase evolution between resonances, more precisely about the presence (absence) of a phase lapse. Keeping in mind that the Green function (Equation 4) is a complex number, a phase lapse with *Π* appears if both the real and the imaginary part change their sign simultaneously. This means that, in turn, the Green function has a *zero* at the phase lapse position, so one has to look for the *zeros* of the Green function.

Mathematically, it easy to show that the Green function in Equation 4 can vanish between consecutive resonances if and only if their numerators have the same sign. We have to keep in mind that the real part of the denominators have opposite signs for consecutive resonances (say, *ε*_*n*_ and *ε*_*n* + 1_), if the Fermi level is between the resonances (i.e., *ε*_*n*_ < *E*_F_ < *ε*_*n* + 1_). Thus, if one wants the sum of two neighboring resonances to vanish, their nominators must have the same sign [10]. Therefore, the existence (or not) of *zeros* in transmittance, or equivalently, a phase lapse of *Π* between resonances can be associated to the same (different) parity of consecutive resonances.

It is important to mention that, even if the simple decomposition in Equation 4 is correct in the limit of low coupling, the fact that the conductance function can be written as a sum of resonances is always true; also, the positions of the Green function’s *zeros* are independent of the coupling strength, as was proven in [10].

We now turn to the interferometer geometry. In [26] (where the same system is considered as here, namely a QD inserted in a closed interferometer), it was shown that the conductance can be expressed in terms of two effective Green functions for the embedded dot and for the two sites ring:

(5) $$G=\frac{2e^{2}}{h}4\Gamma^{2}{\left|{\tilde{G}}_{\text{αβ}}^{R}+\tau^{2}\underset{\gamma ,\gamma^{\prime }=\alpha ,\beta }{\sum }{\tilde{G}}_{\alpha \gamma }^{R}{\tilde{G}}_{i_{\gamma }i_{\gamma^{\prime }}}^{D}{\tilde{G}}_{\gamma^{\prime }\beta }^{R}\right|}^{2},$$

where ${\tilde{G}}^{D}(E)={\left(E-H^{D}+\Sigma^{D}\right)}^{-1}$ and $\Sigma^{D}=-\tau^{2}\sum_{\gamma ,\gamma^{\prime }=\alpha ,\beta }|i_{\gamma }\rangle {\tilde{G}}_{\gamma \gamma^{\prime }}^{R}\langle i_{\gamma^{\prime }}|$. For our simplified model, we can calculate straightforwardly (we assumed the diagonal energies in the ring sites to be *zero*, and also *E*_F_ = 0):

(6) $$\begin{array}{lll} \;{\tilde{G}}_{\text{αβ}}^{R} & ={\tilde{G}}_{\text{βα}}^{R}=-\tau_{\text{αβ}}/(\tau_{\text{αβ}}^{2}+\Gamma^{2})=:g_{1},\;\\ \;{\tilde{G}}_{\text{αα}}^{R} & ={\tilde{G}}_{\text{ββ}}^{R}=-\mathrm{i\Gamma}/(\tau_{\text{αβ}}^{2}+\Gamma^{2})=:g_{2},\; & \; \end{array}$$

Finally, using the notations *τ*_*nα*_ := *τ* *Ψ*_*n*_(*i*_*α*_) and *τ*_*nβ*_ := *τΨ*_*n*_(*i*_*β*_) one obtains

(7) $$\Sigma_{n}^{D}=-\underset{\gamma \gamma^{\prime }=\alpha ,\beta }{\sum }\tau_{\mathrm{n\gamma}}\tau_{n\gamma^{\prime }}{\tilde{G}}_{\gamma \gamma^{\prime }}^{R},$$

for the self-energy corresponding to the dot level *ε*_*n*_. Introducing in Equation 5 the required expressions which one arrives at:

(8) $$\begin{array}{c} G=\frac{2e^{2}}{h}4\Gamma^{2}{\left|g_{1}+\underset{n}{\sum }\frac{\left(\tau_{\mathrm{n\alpha}}^{2}+\tau_{\mathrm{n\beta}}^{2})g_{1}g_{2}+\tau_{\mathrm{n\alpha}}\tau_{\mathrm{n\beta}}(g_{1}^{2}+g_{2}^{2}\right)}{-E_{n}-(\tau_{\mathrm{n\alpha}}^{2}+\tau_{\mathrm{n\beta}}^{2})g_{2}-2\tau_{\mathrm{n\alpha}}\tau_{\mathrm{n\beta}}g_{1}}\right|}^{2}. \end{array}$$

For well-separated resonances (meaning that the distance between resonances exceeds the resonances width), the summation symbol *Σ*_*n*_ can be placed in front of the square modulus. Then inside the modulus, we bring the terms to the same denominator as follows:

(9) $$G=\frac{2e^{2}}{h}4\Gamma^{2}\underset{n}{\sum }{\left|\frac{-g_{1}E_{n}+\tau_{\mathrm{n\alpha}}\tau_{\mathrm{n\beta}}(g_{2}^{2}-g_{1}^{2})}{-E_{n}-(\tau_{\mathrm{n\alpha}}^{2}+\tau_{\mathrm{n\beta}}^{2})g_{2}-2\tau_{\mathrm{n\alpha}}\tau_{\mathrm{n\beta}}g_{1}}\right|}^{2}$$

By replacing *g*_1_ and *g*_2_ in Equation 9, one can convince himself after some manipulation that the conductance can be expressed as a sum of Fano resonances [27]:

(10) $$G=A\underset{n}{\sum }\frac{{\left(\varepsilon_{n}+q_{n}\right)}^{2}}{\varepsilon_{n}^{2}+1}$$

with the following notations

(11) $$\begin{array}{lll} \;q_{n} & =\tau_{\mathrm{n\alpha}}\tau_{\mathrm{n\beta}}\frac{\left(\frac{\tau_{\text{αβ}}}{\Gamma }-\frac{\Gamma }{\tau_{\text{αβ}}}\right)}{\left(\tau_{\mathrm{n\alpha}}^{2}+\tau_{\mathrm{n\beta}}^{2}\right)}\;,\;A=\frac{2e^{2}}{h}4\Gamma^{2}\frac{\tau_{\text{αβ}}^{2}}{{\left(\tau_{\text{αβ}}^{2}+\Gamma^{2}\right)}^{2}}\;,\; & \;\\ \;\varepsilon_{n} & =\frac{\tau_{\text{αβ}}^{2}+\Gamma^{2}}{\Gamma (\tau_{\mathrm{n\alpha}}^{2}+\tau_{\mathrm{n\beta}}^{2})}E_{n}-2\frac{\tau_{\mathrm{n\alpha}}\tau_{\mathrm{n\beta}}\tau_{\text{αβ}}}{\Gamma (\tau_{\mathrm{n\alpha}}^{2}+\tau_{\mathrm{n\beta}}^{2})}.\; \end{array}$$

The above expression for *q*_*n*_ is the main result of our paper, which shows [keeping in mind that *τ*_*nα* _*τ*_*nβ*_ = *τ*^2^*Ψ*_*n*_(*i*_*α*_)*Ψ*_*n*_(*i*_*β*_)] that the parity information can be extracted from the sign of the Fano parameter.

The sign of the quantity $\left(\frac{\tau_{\text{αβ}}}{\Gamma }-\frac{\Gamma }{\tau_{\text{αβ}}}\right)$ is not important because it is independent of the resonance index *n*, but what is important is the relative signs of *q* (or of the parities) for different resonances. One should mention that in the presence of a magnetic flux applied on the AB interferometer, the Fano parameter becomes complex [26]; however, for the purpose of extracting phase information, one does not need a magnetic flux.

At this point we make use of the equivalence parity ⇔phase lapse described in the beginning of this section (following [10]), and now we can finally conclude that the existence (or not) of phase lapses between consecutive resonances can simply be decided by inspecting the sign of the Fano parameters for consecutive resonances (that are experimentally observable: *q* > means that the Fano line forms first the dip and then the peak, while for *q* < 0 the situation is the opposite). This equivalence was suggested in [12], by numerical results, but the analytical proof was missing.

The quantities *τ*_*nα*(*β*)_ (defined in the paragraph above Equation 7) are called effective coupling parameters, so one can also say that the Fano parameter *q* carries information about the relative sign of the effective coupling parameters.

**Figure 2:**
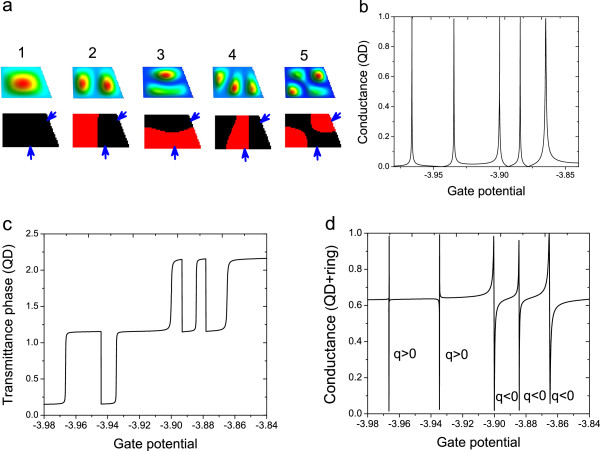
**The first five eigenfunctions of an arbitrary-shaped QD.** (**a**) The sign distribution of the eigenmodes are plotted below the amplitudes (by convention, we attribute the color black to the sign ‘+’ and the color red to the sign ‘-’). The small blue arrows indicate the position where the leads will be connected. Transmittance amplitude (**b**) (which is proportional to the conductance) and phase (**c**), respectively, for the same QD with the leads connected as mentioned. The conductance is in units 2e^2^/h and the phase is in *Π* units. (**d**) The corresponding Fano lines which would result by inserting the same QD in an interferometer. Near each Fano resonance we wrote the sign of the Fano parameter.

The analytical results derived in this section are illustrated by a numerical calculation in Figure 2, for the case of an arbitrary-shaped quantum dot. In Figure 2a, we plot the amplitudes and phases of the first five eigenmodes. Since the eigenfunctions can be considered to be real, by ‘phase’ we mean actually the sign of the wave function. By convention, let us assume that the color black in the figure corresponds to the sign ‘+’ and red to the sign ‘-’.

If our quantum dot is connected to leads (the position of the leads is indicated by the small blue arrows in Figure 2a), the resulting transmittance is plotted in Figure 2b, and the transmittance phase in Figure 2c. Furthermore, if the dot is placed in one arm of an interferometer, one obtains Fano lines - plotted in Figure 2d - with both positive and negative sign of the Fano parameter. The first two Fano resonances in Figure 2d are very narrow, but indeed they form the dip first and then the peak, meaning they have positive Fano parameter (*q*>0).

As expected, the numerical results confirm the analytical results presented in this section. The resonance numbers 1 and 2 have the parity ‘+’ (meaning that the leads are connected to points where the eigenmodes have the same signs) and numbers 3, 4, and 5 have the parity ‘-’ (the corresponding eigenmodes have different signs in the contact sites). As it was shown earlier in this section between resonances of the same parity, the phase exhibits a *Π* lapse; while between resonances of different parity, the phase remains constant. On the resonances themselves, the phase evolves with *Π*, on a gate interval equal to the resonance width.

We notice that the resonances with the same parity generate Fano lines with the same sign for the Fano parameter. It is then clear that by reversing the problem, one can extract the parity and phase behavior from the sign of the Fano parameters of consecutive resonances.

Our calculations have another interesting implication which is described in following: Let us imagine that one contact on the QD is fixed, while the other can be moved on the QD surface. Both contacts are further connected to a ring and the ring to the leads. A variable gate potential applied on the QD generates Fano lines and the sign of the Fano parameter *q* is given by the sign of *Ψ*_*n*_(*i*_*α*_)*Ψ*_*n*_(*i*_*β*_), for each eigenmode. While, as mentioned, the position of one contact is varied on the QD surface, the Fano parameter will only change sign if the ‘mobile’ contact crossed a nodal line (i.e., reached a zone with an opposite sign of the respective eigenmode). Therefore, one can map the sign (phase) distributions of the eigenmodes. The phase mapping of the eigenmodes on a 2D surface was considered to be possible only for isospectral shapes, as proposed by Moon et al. [28] (see also [29,30]). In pairs of isospectral shapes, the wave functions can be expressed in terms of each other, which brings a supplementary information, allowing to extract the phase distributions, if the amplitude distributions are known. Our proposed scheme makes use of the Fano effect and it is not necessary for the shape (for which the phase distributions are extracted) to be isospectral. The drawback however, comes from the fact that present experimental technics do not allow - to our knowledge - to connect the leads in arbitrary points on a QD surface; rather, the position of the leads is fixed as they are defined by lithography at the same time with the QD, for semiconductor QDs.

## Conclusions

We have shown that important transmittance phase information can be extracted using the so-called ‘closed’ mesoscopic interferometers, in spite of the fact that multiple interferences are in this case allowed, and it was generally believed that a correct phase extraction would be blurred.

If a gate potential is applied on a QD inserted in one arm of a mesoscopic ring (i.e., a closed interferometer), the result in conductance would be the asymmetric Fano resonances. We presented detailed calculations directly relating the sign of the Fano parameter - for a dot + ring system - with the behavior of transmission phase between resonances of the bare dot. In-phase (or out-of-phase) neighboring resonances of the dot correspond to the Fano line shapes with the same (opposite) sign for the Fano parameter. A lapse with *Π* of the phase is present (or absent) between in-phase (or out-of-phase) resonances.

As mentioned, the important implication is that the famous ‘phase lapse’ problem can be addressed in closed interferometers simply by inspecting the sign of consecutive Fano parameters. If we apply this interpretation to some existing experiments [24,25] which focused on the Fano effect in closed interferometers (but did not discuss the phase problem), a non-universality of the phase-lapse aspect can be suggested, as one can notice Fano lines in the same but also in the opposite sign of the Fano parameter. This was first suggested in [12], but the analytical proof was missing, and we provided it in the ‘Results and discussion’ Section of this work. This is the main result of our paper and should be important because in the absence of analytical formulas, the numerical results can always be considered as sample-dependent.

An interesting implication would be that the Fano effect can also be used to map the phase distribution of the eigenmodes on a mesoscopic shape (previously, the mapping of the wave function phase was proposed for isospectral shapes [28-30]). This is possible because the Fano parameter carries information also about the parity of the resonances.

The results presented in this paper are obtained for the non-interacting model and they are either reproduced from existing literature (for completeness), or new proofs are provided where they were missing. The case with interaction presents an increased complexity, and a deep analysis will be the subject of a future work (in [31] we present some results on a toy interacting model). Such an analysis should be necessary, since the electronic correlations are expected to play an important role in the phase problem (see e.g., [3,14-17,20,21]).

We hope that our work will motivate new kind of experiments, combining both closed and open interferometers, in order to bring further clarification in the long-debated phase lapse problem.

## Competing interests

The authors declare that they have no competing interests.

## Authors’ contributions

The authors declare equal contributions to this paper. All authors read and approved the final version.
